# Wnt affects symmetry and morphogenesis during post-embryonic development in colonial chordates

**DOI:** 10.1186/s13227-015-0009-3

**Published:** 2015-05-01

**Authors:** Alessandro Di Maio, Leah Setar, Stefano Tiozzo, Anthony W De Tomaso

**Affiliations:** School of Bioscience, University of Birmingham, Edgbaston, Birmingham, B19 2TT UK; Molecular Cellular and Developmental Biology, University of California Santa Barbara, Santa Barbara, CA 93106 USA; CNRS, Sorbonne Universités, UPMC Univ Paris 06, Laboratoire de Biologie du Développement de Villefranche-sur-mer, Observatoire Océanographique, 06230 Villefranche-sur-mer, France

**Keywords:** Wnt, Polarity, Symmetry, Asexual development, Chordates

## Abstract

**Background:**

Wnt signaling is one of the earliest and most highly conserved regulatory pathways for the establishment of the body axes during regeneration and early development. In regeneration, body axes determination occurs independently of tissue rearrangement and early developmental cues. Modulation of the Wnt signaling in either process has shown to result in unusual body axis phenotypes. *Botryllus schlosseri* is a colonial ascidian that can regenerate its entire body through asexual budding. This processes leads to an adult body via a stereotypical developmental pathway (called *blastogenesis*), without proceeding through any embryonic developmental stages.

**Results:**

In this study, we describe the role of the canonical Wnt pathway during the early stages of asexual development. We characterized expression of three Wnt ligands (Wnt2B, Wnt5A, and Wnt9A) by *in situ* hybridization and qRT-PCR. Chemical manipulation of the pathway resulted in atypical budding due to the duplication of the A/P axes, supernumerary budding, and loss of the overall cell apical-basal polarity.

**Conclusions:**

Our results suggest that Wnt signaling is used for equivalent developmental processes both during embryogenesis and asexual development in an adult organism, suggesting that patterning mechanisms driving morphogenesis are conserved, independent of embryonic, or regenerative development.

**Electronic supplementary material:**

The online version of this article (doi:10.1186/s13227-015-0009-3) contains supplementary material, which is available to authorized users.

## Background

A major question in developmental biology is how symmetry in the early embryo is broken and body axes established to guide further development. These essential patterning mechanisms mainly occur during post-embryonic developmental process, such as metamorphosis or the regeneration of lost or damaged structures. However, the timing and developmental pathways driving these early patterning processes during post-embryonic development are not well understood. Embryogenesis, as well as organs or body regeneration, is a process where a tight control of cell-cell regulation is fundamental to establish Cartesian axes and eventually to lead to body patterning or re-patterning [1]. One of the key mediators of cell-cell signaling during embryogenesis is the highly conserved Wnt signaling pathway [2-7]. The Wnt family encompasses a set of secreted glycoproteins involved in variety of developmental processes in both bilaterian and pre-bilaterian metazoans. Members of the Wnt family regulate cell-cell interactions and affect multiple cell and tissue behaviors including proliferation, differentiation, migration, and polarity [8-9]. Wnt proteins are key players in at least three signaling pathways: the planar cell polarity (PCP); the Wnt/calcium pathway (the non-canonical pathways), which do not involve β-catenin [10-13]; and the canonical Wnt pathway, which involves the nuclearization of β-catenin [6,14-16]. The canonical signaling pathway is the most studied to date and plays a primary role on early axes determination, patterning, and specification in embryos of most metazoans [2,6,7,17]. Particularly, the establishment of the axial polarity in multicellular organisms has been associated with a localized occurrence of maternal β-catenin protein and *wnt* gene expression domains, specific for the dorsal and caudal pole of a wide variety of metazoan [7].

The same spatial cues regulated by the Wnt ligands during embryogenesis also be used in post-embryonic developmental pathways, and Wnt has been shown to play a role during metamorphosis [18], during regeneration following injury [4,19], and in organisms that propagate asexually, including cnidarians [2,20,21], annelids [22], and ascidians [23].

Ascidians (subphylum Tunicata) are the closest relatives to vertebrates that can regenerate their entire bodies [24,25] and provide a chordate model organism to dissect the molecular mechanisms of the regenerative process. Ascidians are chordates, and embryogenesis results in a tadpole larva which, following a relatively short free-swimming phase, settles and metamorphoses into a sessile, filter-feeding juvenile form during which most of the chordate features are lost (benthic-phase). Whereas individuals of solitary ascidians grow in size and eventually reach sexual maturity, colonial forms grow via repeated rounds of asexual reproduction: following metamorphosis, a single founder individual (called oozooid) propagates asexually, eventually creating a colony of several to hundreds of individuals (called zooids), which are indistinguishable and genetically identical from the founder oozooid [26].

The species *B. schlosseri* is a reference model for the study of chordate asexual development and regeneration as it undergoes a cyclical, rapid (7 day), and coordinated budding process that can be easily visualized [27,28]. Asexual development in *Botryllus* is called blastogenesis [27] and is a complex process that couples regeneration and turnover of zooids. In a colony of *B. schlosseri*, three asexual (blastogenic) generations coexist in a spatially organized structure of 2 to 20 individuals called a system. The center of the system contains the zooids, which are filter-feeding and sexually reproducing adult individuals, and they are joined laterally by primary buds, which are completing development. Lastly, the primary buds are connected in turn to the secondary buds (budlets), which are in the early stages of asexual development. The adults have a defined, 1-week lifespan after which they die in a process called *take-over*, whereby all zooids in the colony synchronously undergo apoptosis and the corpses are cleared by phagocytic cells in the blood. The primary buds migrate to the newly open space, open their siphons, and begin their 1-week lifespan as a zooid. Simultaneously, the budlets migrate and become primary buds and initiate the next round of secondary buds. Thus, for a colony to maintain its size, at least one bud (primary or secondary) must complete development each week. A zooid can have up to four primary buds; thus, a colony can asexually expand over the substrate rapidly. Development and apoptosis are synchronized throughout the colony, which can consist of thousands of adult individuals.

Early studies established a method to define the developmental stage of the colony, consisting of a formula of three numbers (for example, 9/8/4) that define well-characterized visual developmental landmarks of each generation (zooid/primary bud/secondary bud) [27]. Under standard laboratory conditions, each stage represents 1 day. A new individual begins as a secondary bud (budlet), which first appears as a thickened disk on the primary bud epidermis (stage 9/7/1) that evaginates and then closes into a blastula-like structure (stages 9/8/2 and 9/8/3, respectively). This epithelial vesicle then undergoes morphogenesis (9/8/4 to 9/8/6), giving rise to the main organs, such as the branchial and peribranchial chambers, gut, and nervous system, and provides a visual anterior-posterior (A/P) and dorsal-ventral (D/V) axes in the developing body. However, stereotypical morphological changes in the budlet (that is, rotations and skewing) prior to organogenesis, demonstrate that there is a specific A/P axis that coincides with that of the parental bud from the initiation of development [29]. The first visual change is the appearance of a morphological polarity in the secondary bud followed by a rotation and elongation of the budlet along the presumptive A/P axis [28,29]. In wild and lab reared colonies, the bilateral asymmetry can be observed by the presence of the gut on the left side, the heart on the right side of the zooid, the development of large testes on the left side, and the production of a larger number of budlets on the right side [30,31].

The aim of this study is to investigate the role of the Wnt signaling pathway during the early stages of the blastogenesis, prior to the initiation of morphogenesis and organogenesis. Among the large Wnt family protein, Wnt2 has been shown to be crucial for organ specification and patterning [32-37] as well as in angiogenesis and endothelial differentiation [38]. Within many species, Wnt5 and Wnt7 are involved in the control of morphogenetic movements and tissue growth and differentiation [7,15,37,39,40]. In this study, we report the expression of these three different Wnt ligands and their potential function in budlet initiation, breaking of symmetry, and axis specification.

## Methods

### Animals

*B. schlosseri* colonies were collected from the harbor in Santa Barbara, CA, spawned and cultured in laboratory conditions at 18°C to 20°C [41]. Colonies were developmentally staged-matched based on blastogenic stage cycles [27].

### Isolation of *B. schlosseri* Wnts and phylogenetic analyses

*B. schlosseri* Wnts were identified by performing a Basic Local Alignment Search Tool (BLAST) search of our in-house reference transcriptome database (http://octopus.obs-vlfr.fr/public/botryllus/blast_botryllus.php). The whole open reading frame (ORFs) of the *B. schlosseri* othologues of Wnt2B, Wnt9A, and Wnt5A were amplified and cloned into a pGEM-T-EZ vector (Promega), sequenced and used to produce RNA probes for whole-mount fluorescent *in situ* hybridization (FISH).

**Figure 1:**
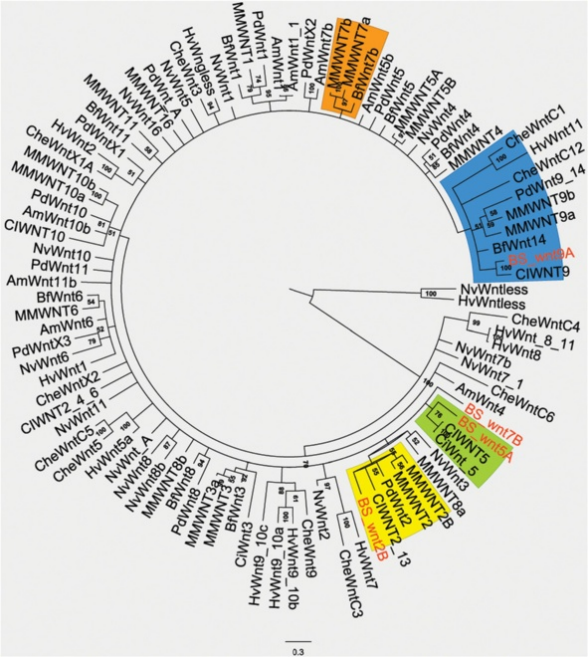
Phylogenetic characterization of four *Botryllus schlosseri* Wnt genes. Bayesian consensus tree of Wnt amino acid sequences constructed as described in the ‘Methods’ section. *B. schlosseri* genes considered are highlighted in bold red whereas clusters with other orthologues are colored (*wnt7* = orange; *wnt5A* = green; *wnt2B* = yellow; *wnt9A* = blue). The tree has been rooted on its midpoint, percentage of posterior probability is showed at every node. Amphi: *Branchiostoma floridae*, Che: *Clytia haemispherica*, MM: *Mus musculus*, Pdu: *Platynereis dumerilii*, Ame: *Apis mellifera*, Nv: *Nemastostella vectensis*, Hv: *Hydra vulgaris*, Ci *Ciona intestinalis*, Bs: *Botryllus schlosseri*.

Multiple sequence alignments were built using MUSCLE [42], and conserved blocks have been selected using GBLOCK and trimmed manually. Bayesian trees were built via the Markov chain Monte Carlo method by using MRBAYES [43], adopting a Jones-Taylor-Thorton (JTT) mathematical method substitution matrix. Two Markov chains were run each containing 20,000,000 Monte Carlo steps. One out of every 100 trees was saved. The trees obtained on each run were meshed, and the first 25% were discarded as burn-in. Marginal probabilities for internal branches were taken as measures of statistical support. Only the consensus tree is shown in Figure 1. All the trees are available upon request.

### *In situ* hybridization

Whole-mount FISH was performed with digoxigenin (DIG)-labeled probes as previously described [44]. Specific sense and antisense probes for *wnt2B*, *wnt5A*, and *wnt9A* were synthesized from PCR products using the relative clones coding for 844 bp, 722 bp, and 887 bp regions of the respective genes. Primers used to synthesize probes were 5′-gttgtgtgcgtcacctgttc-3′ and 5′-tttgggtccgttttgggtga-3′ for *wnt2B*; 5′-tcgctagcatcgcctcgtgc-3′ and 5′-aaagtgcgcaggccctcgag-3′ for *wnt5A*; 5′-cgagagatggaactgcacga-3′ and 5′-aagggagactgtgcgacaag-3′ for *wnt9A*. TSA-plus detection (NEL753001KT PerkinElmer, Waltham, MA, USA) with cyanine 3 and fluorescein substrates was used in our analysis.

### Imaging

High-resolution confocal images of fixed tissue for *in situ* hybridization were acquired using an Olympus1000 Spectral Confocal Microscope (Tokyo, Japan). Z stacks were obtained at a 1.5-μm step size. Olympus Fluoview acquisition software (Tokyo, Japan), Fiji (NIH, Baltimore, MD, USA) and Imaris (Bitplane, Zurich, Switzerland) software were used to acquire and reconstruct z-series images into standard deviation projections at the NRI Microscopy Facility. Live imaging of control and drug-treated colonies were carried out using a Leica MZ16FA epifluorescent stereomicroscope. Images from live animals were also used for morphometrical analysis by using ImageJ (NIH, Baltimore, MD, USA) plug-ins. Figure panels were mounted using FigureJ (Image J) and Photoshop (Adobe, San Jose, CA, USA).

### Quantitative RT-PCR

Quantitative RT-PCR (qRT-PCR) was carried out using a LightCycler 480 II (Roche, Basel, Switzerland) using LightCycler DNA Master SYBR Green I detection (Roche, 12015099001). The thermocycling profile was as follows: 5 min at 95°C, 45 cycles of 95°C for 10 s, 55°C for 10 s, and 72°C for 10 s. Single systems of *B. schlosseri* at stages 9/7/1, 9/8/2, and 9/8/3 were quickly flash frozen in liquid nitrogen, and cDNA pools were generated from messenger RNA (mRNA) extracted from them. Primers for *wnt2B* 5′-gaaggtcggaacagcggtat-3′ and 5′-agcttgtttcagccctgtgt-3′ which amplified a 215 bp fragment, *wnt5A* primers 5′-caccagttcagtgaccatcg-3′ and 5′-gctgtcacaaaagcctgtca-3′ which amplified a 149 bp fragment, and *wnt9A* primers 5′-caaatgccatggtgtgtcgg-3′ and 5′-gcgacgactttcccaacaac-3′ which amplified a 132 bp fragment were used in our analysis. All gene expression data was normalized to elongation factor 1-*α* (EF1-*α*) as a reference gene and reported as relative expression using the 2^-∆∆Ct^ method [45]. Each experiment was analyzed in triplicate from different genotypes (*n* = 3) and multiple systems (*n* = 8).

### Chemical treatments

To determine the effects of blocking GSK-3β on A/P axis formation, adult and juvenile colonies of *B. schlosseri* were treated with two known Wnt agonists (GSK-3β inhibitors): alsterpaullone (APL) (Sigma, MO) and lithium chloride (LiCl). Colonies of two to three systems each were soaked for 14 to 18 h in jars of 400 mL and then returned to normal filtered sea water (FSW) in our mariculture facility. Incubations were carried on for 10 to 14 days in order to ensure that two full blastogenetic cycles were included in the treatments. APL was dissolved in dimethyl sulfoxide (DMSO) and diluted in FSW up to a final concentration of 0.5 μM. Low doses (0.01 μM and 0.1 μM) of APL did not give any specific phenotype whereas higher concentrations (1 μM and 5 μM) were lethal after 1 day of incubation. Lithium chloride was dissolved in dd-water and diluted in FSW up to a final concentration of 28 mM. Control colonies were selected among genetic clones of the treated samples and incubated with a comparable DMSO concentration for the same length of time. At the onset of the experiment, the blastogenic stages of the treated and the control colonies were synchronized.

### Transmission electron microscopy and histology

After anesthetization with MS-222 (Tricaine; ethyl 3-animobenzoate methanesulfonate salt) for about 5 to 7 min in FSW, animals were fixed with 1.5% glutaraldehyde buffered with 1.6% NaCl and 0.2 M sodium cacodylate (pH = 7.2) on ice. Following fixation, they were soaked overnight in 0.1 M sodium cacodylate buffer and the day after rinsed with fresh buffer and post-fixed in cold with 2% OsO_4,_ buffered with 0.2 M sodium cacodylate. After washing with H_2_O, samples were *enbloc* stained in uranyl acetate, dehydrated and embedded in Epon. In order to keep a correct orientation of the animal, samples were first flat embedded on aluminum dishes and then cut with a jeweler saw and re-embedded in molds with an orthogonal orientation. Thin sections (90 nm) were stained in uranyl acetate and lead citrate and examined in a JEOL 1234 Electron microscope (Tokyo, Japan). Out of the same EM samples, thick sections (150 nm) were stained with a solution containing toluidine blue and methylene blue and then observed on a compound microscope to verify the correct orientation.

## Results

### Identification of the Wnt genes in *B. schlosseri*

Using a *de novo* assembled transcriptome database (http://octopus.obs-vlfr.fr/public/botryllus/blast_botryllus.php), we identified several *B. schlosseri wnt* genes among which four sequences showed strong similarities (*e*-value <1*e*^−40^) with orthologs in other species (Additional file 1: Table S1). The predicted amino acid coding sequences were aligned with other metazoans Wnts, and a multiple phylogenetic reconstruction showed orthologies for three out of the four selected sequences (Figure 1). Whereas *B. schlosseri* Wnt2B, Wnt5A, and Wnt9A showed a statistically significant orthology with *wnt* genes from other ascidians and vertebrates, we did not have enough resolution to establish the orthology of *Botryllus* Wnt9. Therefore, we did not include the latter on the analysis of the expression patterns.

### Characterization of spatio-temporal Wnt expression during early blostogenetic cycle

**Figure 2:**
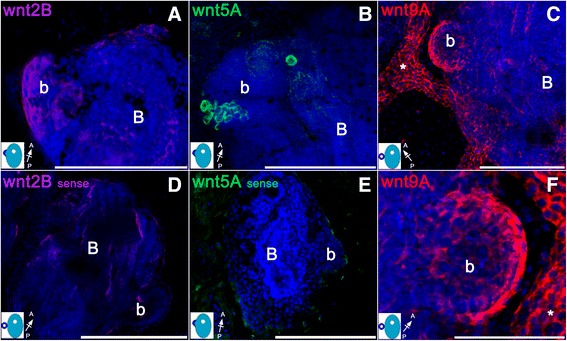
Spatio-temporal Wnt expression during early blostogenetic cycle. Expression patterns of *wnt2B* (magenta), *wnt5A* (green), and *wnt9A* (red) in *Botryllus schlosseri* buds processed for whole-mount FISH. All images are obtained by an average projection of the confocal z-stack image. The small sketch on the lower left corner of each panels represents the blastogenic stage of the buds shown (blue = secondary bud; light blue = primary bud), and the small arrow indicated their A/P orientation. **(A)** Confocal image of a primary **(B)** and secondary (b) buds at stage 9/8/2 showing expression of *wnt2B* on the emerging budlet (b) early in the blastogenesis. **(B)** *wnt5A* mRNA is expressed in immature germline cells within the gonadal blastema at stage 9/8/2. **(C)** At the same developmental stages considered, *wnt9A* is expressed predominantly within the secondary bud (b). Note that the epithelial wall of the vasculature (asterisk) also does express *wnt9A.* **(D)** Sense probe used on untreated buds as negative control for *wnt2B*. **(E)** Confocal image of wild type animal stained with a sense probe used as negative control for *wnt5A*. **(F)** Enlargement of the secondary bud (b) in panel **C** showing *wnt9A* expression. BARS: 200 μm in **A**, **B**, **C**, **E**. 100 μm in **D** and **F**.

**Figure 3:**
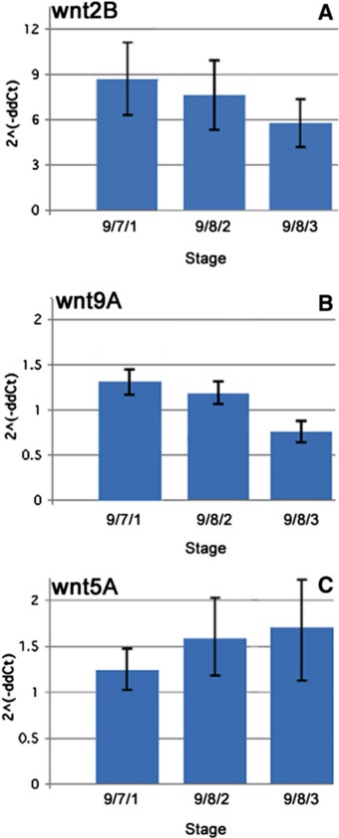
Temporal expression profile of Wnt candidate genes in colonies at early blastogenic stages. Quantitative reverse transcriptase PCR (qRT-PCR) expression analysis of Wnt ligands Wnt2B **(A)**, Wnt9A **(B)**, and Wnt5A **(C)** within the first three stages of the blastogenic life cycle. The ordinate values are relative expression levels normalized to the expression level of Ef1-*α* in 9/7/1 stage according to the 2^-∆∆Ct^ (see ‘Methods’ section) and displayed in arbitrary units.

To assess the putative role of Wnt proteins in early developmental processes, we characterized gene expression patterns of *B. schlosseri wnt* orthologs during the first 3 days of secondary bud development by FISH, prior to the onset of morphogenesis (from stage 9/7/1 to 9/8/3). As shown in Figure 2, *wnt2B* and *wnt9A* transcripts are expressed in the emerging secondary bud (Figure 2A, C, and F). In contrast, *wnt5A* seems to be expressed exclusively in a cell mass found in the mesenchymal space of the primary bud, which is part of the developing gonads (Figure 2B) (Langenbacher *et al*. [44]). We also found *wnt9A* expression in the epithelial wall of the vasculature (asterisk in Figure 2C) within all stages considered. The distribution of the *wnt9A* and *wnt2B* in the bud primordium appeared uniform in animals from 9/7/1 to 9/8/3 stage of blastogenesis whereas no signal was detected in the primary buds (Figure 2A and C). Furthermore, results from quantitative RT-PCR (Figure 3) analysis performed on cDNA pools from single systems confirmed the presence of *wnt* revealing the expression patterns of the ligands during early stages of the blastogenesis. *Wnt2B* and *wnt9A* showed similar trends of expression throughout these time periods, with no significant variability between stage 9/7/1 and 9/8/2 (Figure 3A and B). *Wnt5A* showed an opposite trend as in *wnt2B* with a slight increase in expression levels from stage 9/7/1 to 9/8/2 (Figure 3C). These data indicate that there is a trend in the *wnt* expression that follows the early development pattern of *B. schlosseri*.

### Wnt agonists affect early development

In order to study the effect of Wnt signaling during the onset of blastogenesis, colonies were incubated with Wnt pathway agonsists (GSK-3β inhibitors) at take-over (stage 11/8/6), when the budlet starts to form. We noticed multiple phenotypes that correlated with a potential role of the Wnt pathway in early asexual development. The observed phenotypes are summarized in Additional file 2: Figure S1 where solid horizontal lines represent a normal blastogenic development and rough lines the altered development due to the over-activation of the wnt pathway.

### Defects on A/P axis formation upon Wnt agonists treatments

**Figure 4:**
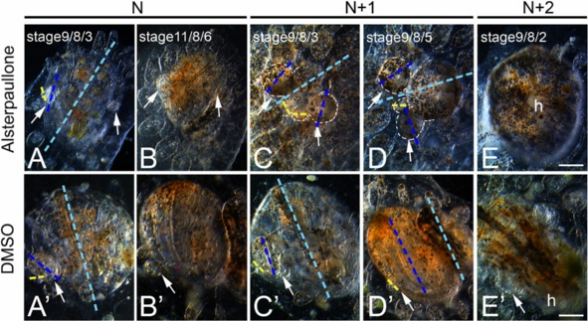
Treatments with Wnt agonists induce changes on the A/P polarity of developing buds. Ventral views of *B. schlosseri* colonies (oozoids) incubated with 0.5 μM APL (top row), and control colonies incubated with DMSO (bottom row). The antero/posterior (A/P) axis of zooid (light blue), primary bud (dark blue), and secondary bud (yellow) are represented in both samples. **(A**-**B)** During the first few days of treatments, no major signs of axis alteration of budlets (arrows) are visible. **(C)** Starting from the second blastogenic cycle (*N* + 1, stage 9/8/3), clear morphological changes are visible throughout the whole animal. Ectopically growing secondary buds (highlighted by white dashed lines) are visible after the first and second blastogenic cycle (days 11 through 16 post incubation) with an altered axis orientation. **(D)** The heavy pigmentation and the different polarity of the budlets persist even approaching the take-over stage (*N* + 1, stage 9/8/5). Note that the budlets axes are now almost perpendicular to the axes of the primary buds compared to a parallel disposition of the control. **(E)** Three weeks later (*N* + 2, stage 9/8/2), primary buds are difficult to recognize due the massive alteration of the overall morphology whereas no secondary buds were found at this point. Now the heart (h) is centrally located compared to the control in **(E’)** situated on the left side. (**A’** through **E’**) Control animals incubated with DMSO did not show any A/P polarity alteration throughout the three blastogenic cycles analyzed. BAR: 200 μm.

Within the first 48 to 72 h of incubation with the Wnt agonists APL and LiCl (cycle N), buds and adult zooids appeared regular (Figure 4A and B). The majority (82%, *n* = 22 colonies) went through take-over normally while other colonies (18%, *n* = 22) followed a shorter (5 days) cycle. However, during the *N* + 1 blastogenic cycle (stage 9/8/3), primary and secondary buds showed a drastic change of their A/P polarity together with increased pigmentation (Figure 4C). Although at the time of the incubation adult zooids kept a normal A/P axis (Figure 4A and A’), primary buds and budlets at *N* + 1 cycle developed a rounded fashion without the normal symmetry (Figure 4C and D). Later in the cycle (stage 9/8/5), the A/P axis of the budlet drastically changed from being almost parallel to that of the primary bud to being perpendicular to it (Figure 4D). Comparing treated and untreated colonies, we observed a totally different angle between primary and secondary bud axes demonstrating an abnormal development.

In addition, within the first week of treatments, while the control primary bud grew in size, treated colonies (*n* = 11) revealed buds that were about 30% smaller and abnormal (Figure 4D). Both primary and secondary buds kept their atypical, heavily pigmented shape throughout the entire *N* + 1 blastogenic cycle, eventually undergoing take-over. At the *N* + 2 cycle, all the primary buds were reduced in their size to the point of being difficult to observe. Interestingly, at this stage, all the surviving adult zooids were rounded instead of oval-shaped showing abnormal symmetry, with a visible beating heart located centrally (Figure 4E). Notably, no new budlet formation was observed at this stage. All together, these phenotypes represent a clear indication of body patterning alteration and organ misplacement. Control colonies treated with DMSO for the same time frame showed a normal developmental cycle, without any major signs of stress or alteration of the body axes (Figure 4A’ through E’).

### Wnt agonists induce ectopic budlet formation

**Figure 5:**
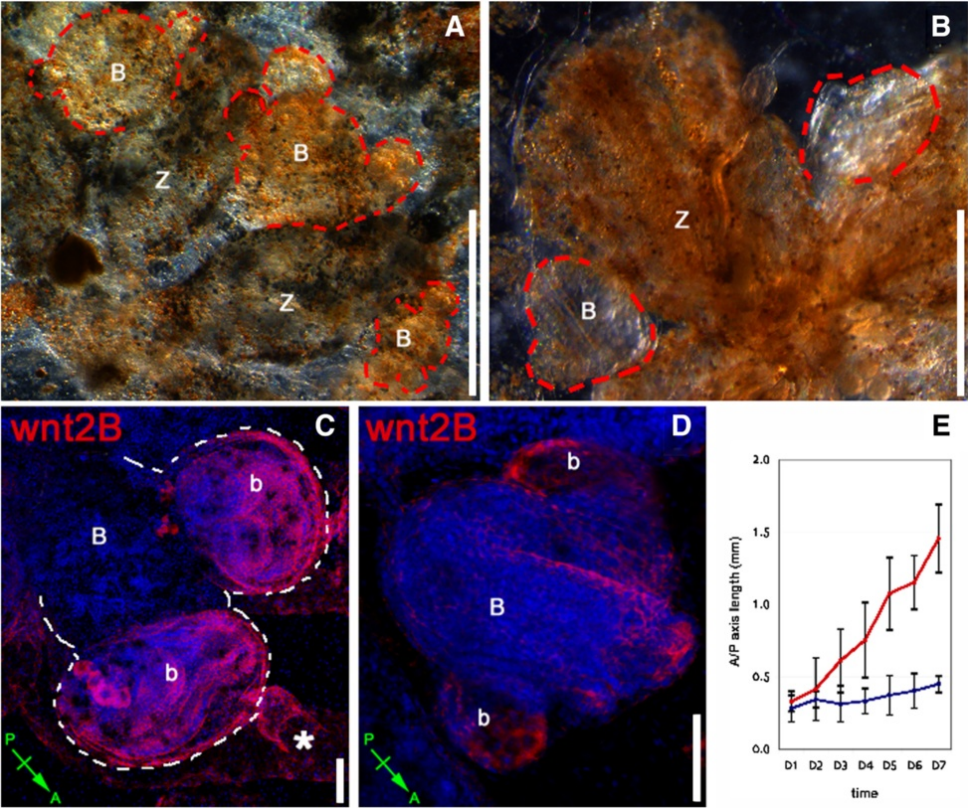
Ectopic secondary bud formation upon Wnt pathway alteration. *B. schlosseri* colonies treated with GSK-3β inhibitor (APL) are massively affected in their overall morphology. **(A)** Ventral views of a colony incubated with Wnt agonists showing ectopic budlets (highlighted with red dashed line) formations from the primary bud (*N* + 1, 9/8/3). **(B)** At stage 9/8/1, control colonies of the same genotype treated with DMSO do not show any morphological alteration. **(C)** Maximum projection of a z-stack of confocal image showing *wnt2B* expression by FISH. Note the high level of expression of *wnt2B* within the two ectopic budlets (b) at stage 9/8/3 (*N* + 1 cycle). The internal vasculature (asterisk) is also labeled with the probe. **(D)** A sub-clone of the animals in **(C)** was used as control for the same FISH experiment. The confocal z-stack image shows *wnt2B* expression located only on the secondary bud (b). Some background staining is visible on the apical side of the primary bud **(B)**, not considered as true fluorescent signal. **(E)** Measurements of the A/P axes of primary buds at *N* + 1 cycle within treated colonies (blue) and control colonies (red). BARS: 1 mm (**A** and **B**); 200 μm (**C** and **D**).

Although during the first days of treatments with APL we did not observe any major signs of morphology alteration, 10 days after the beginning of the drug incubation (9/8/3 stage - *N* + 1 cycle), colonies showed increased pigmentation and abnormal organization of ampullae together with multiple ectopic secondary buds that were sometimes irregular in shape and size (outlined in red in Figure 5A). At that point, primary buds were smaller and rounded compared to control animals (Figure 5B). The source of the ectopic budlets was the first individual to receive treatment, suggesting that Wnt may be involved in budlet initiation or in early steps of budlet evagination.

A similar phenotype was observed in colonies treated with 28 mM of LiCl. During the *N* + 1 blastogenic cycle, primary buds showed a circular profile with increased pigmentation and also multiple ectopic secondary buds that were irregular in shape and size compared to the controls (data not shown).

Interestingly, *in situ* hybridization experiment performed in treated animals revealed a more abundant expression of *wnt2B* throughout the ectopic secondary buds and vasculature walls (Figure 5C). On the contrary, control animals showed normal expression only in the emerging budlet (Figure 5D), demonstrating again that an alteration of the Wnt signaling pathway induces an abnormal development early in the blastogenesis.

Morphometric analysis (*n* = 20) of the primary bud’s A/P axes at *N* + 1 cycle (Figure 5E) confirmed the drastic change in development of the budlet during its late first blastogenic cycle (end of the *N* cycle in Additional file 2: Figure S1). This result further corroborates the strong phenotype observed upon Wnt agonist treatments, showing a smaller budlet size within the first 2 weeks of treatments.

### Over-activation of the Wnt pathway with agonist triggers A/P axis duplication

**Figure 6:**
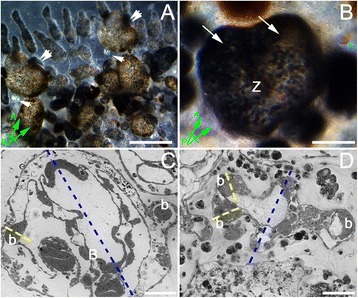
Wnt pathway over-activation induces A/P axes duplication and elongation. **(A)** Upon chemical treatments with Wnt pathway agonist (LiCl), in some cases, single individuals within a colony showed signs of axes duplication (double arrows). At *N* + 2 cycle, next to the adult bifurcated individual, only primary buds are visible (single arrows). **(B)** Dorsal view enlargement of one of the zooids in **(A)** showing a double oral siphon. **(C)** Toluidine blue staining of semi-thick sections (150 μm) from control colony indicating the A/P axes orientation on primary (B - blue) and secondary (b - yellow) buds. **(D)** The same preparation on drug-treated colony shows bifurcated peribranchial epithelium within the budding region. BAR: 1 mm **(A)**; 200 μm **(B)**; 500 μm (**C** and **D**).

In addition to ectopic budlet formation and changes in A/P polarity, we also observed a duplication of the main A/P axis in either APL- or LiCl-treated animals. In about 20% of cases (*n* = 22) at *N* + 2 cycle, the surviving zooids showed axes duplication with two oral and one atrial siphon (Figure 6A and B). Histological sections of control and drug-treated colonies (Figure 6C and D, respectively) show a bifurcated peribranchial epithelia in the presumptive budding region demonstrating that duplicated axes appeared during budlet initiation, at *N* + 1 cycle.

Due to the severe effects of the drugs on the body patterning, we were not able to follow any development after the *N* + 2 cycle. Almost 90% (*n* = 22) of the treated colonies died after the third blastogenic cycle post incubation. The few colonies that did survive were difficult to analyze due the abnormal shape, thickness of the tunic, and extremely heavy pigmentation.

Taken together, our *in vivo* observations clearly show a strong perturbation of the A/P axis throughout the blastogenic cycle. Treatments with APL (or LiCl) which inhibit the GSK-3β activity not only induce ectopic budding but also compromise the A/P polarity of the colony.

### Cellular phenotypes following Wnt activation

**Figure 7:**
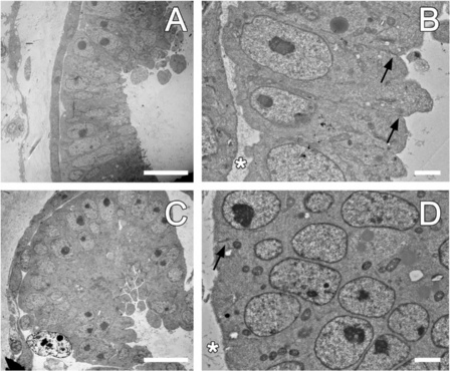
Modification of the inner epithelial cells upon activation of Wnt. At the ultrastructural level, alteration of the Wnt pathway induces polarity changes of cells composing the thickening of the atrial epithelium of the inner vesicle of the primary bud. The normal pattern of cells oriented toward the outer side of the budlet **(A)** is mostly lost in drug-treated colonies **(C)**. Particularly, in drug incubated animals **(D)**, epithelial cells lose their nuclei orientation compared to the control animals **(B)**. The collagen content (asterisks) in treated samples is thinner compared to the non-treated tissues. Shallow intercellular junctions (arrows) are visible in both preparations showing no structural differences. BAR: 10 micron (**A** and **C**); 1 micron (**B** and **D**).

The initial stage of budlet development is a thickening of the peribranchial mono-layered epithelium of the primary bud, which evaginates, pushing out the overlaying epidermal epithelium, then pinches off to form a double layer vesicle [27]. At this stage, the inner layer folds into a sealed vesicle composed of thick cylindrical epithelial cells organized in a palisade layer. Cells are polarized showing a large nucleus located at the apical side, with a large nucleolus and mitochondria scattered in the cytoplasm (Figure 7A and B) whereas cells in the external epithelium do not show this morphology.

Drug-treated animals exhibited a significantly different secondary bud morphology. Thin sections of samples treated with APL and LiCl at stage 9/8/2 (*N* + 1 cycle) showed abnormal budlet onsets composed of a cluster of cells without any defined shape, and many of them lack orientation (Figure 7C and D). Ultrastructural observations showed that epithelial cells lost their cylindrical shape and became spherical, with reduced cytoplasm and large nuclei (Figure 7D). The whole epithelium was composed of cells clustered together by short and shallow intercellular junctions (arrows in Figure 7B and D). The apical plasmalemma was covered by a thick glycocalyx as similar to the control tissue whereas no major differences were found in the basal lamina structure covering the apical side facing the blood vessel (Figure 7A and C). Here however, the collagen content was lighter and thinner compared to the non-treated tissues (asterisks in Figure 7B and D). Compared to untreated animals, the nucleus (mostly centrally located) was smaller but still contained a large nucleolus and chromatin granules (Figure 7B and D). The overall organization of cells also changed, showing scarce mitochondria content located close to the nuclear membrane. Altogether, our ultrastructural observations lead to the assumption that Wnt over-activation induces tissue alteration that might be related to changes in polarity of individual cells. In turn, altered cell structure would cause the observed macroscopic symmetry phenotypes during morphogenesis.

## Discussion

The role of the Wnt signaling pathway has been widely studied in many different Metazoans, mainly during embryogenesis and during early morphogenesis [5,12,14,46]. In *Xenopus* and zebrafish, the wnt/β-catenin pathway is involved in organizer formation, which subsequently sets up the dorsoventral axis [47,48] while in sea urchins, β-catenin regulates the patterning of the antero-ventral axis so that the vegetal end forms endoderm [15]. Similarly, in the ascidian *Ciona intestinalis*, the localization of β-catenin via canonical Wnt pathway controls endoderm formation and represents the first molecular marker of embryo polarization [16]. The canonical Wnt pathway has also been shown to be involved in gastrulation and germ layer specification during cnidarian embryogenesis [49,50]. The involvement of the Wnt pathway during post-embryonic development (for example, metamorphosis) has also been demonstrated in experiments blocking the activity of GSK-3β, which induces the formation of ectopic axial structures during development [51-53]. We show here that a similar phenomenon occurs in *B. schlosseri* where the alteration of the wnt signaling pathway via GSK-3β inhibition results in drastic changes on body patterning during secondary bud formation.

### Co-option of Wnt ligands during blastogenesis

We have found temporal and spatial expression of Wnt ligands (*wnt5A*, *wnt2B*, and *wnt9A*) within the developing budlets during the early asexual development, and observed phenotypes following chemical-induced Wnt over-activation. Both fluorescent *in situ* hybridization and quantitative RT-PCR showed spatio-temporal expression patterns from blastogenic stages 9/7/1 to 9/8/3, suggesting that *wnt* mRNA is present in the early development of *B. schlosseri*. Indeed, during development, strong *wnt2B* and *wnt9A* expressions were found in the thickening of the peribranchial epithelium, the expanding region of the primary bud confirming that wnt is expressed during blastogenesis. Interestingly, the presence of *wnt9A* on the vasculature wall may allow us to speculate that Wnt signaling might be involved in angiogenesis and vascular development. The expression of *wnt5A* is almost identical to the expression of *vasa* within the same cluster of germline stem cells (GSCs) [44] suggesting that *wnt5A* mRNA may have a role in the germline formation. Alternatively, the expression of *wnt5A* that we detected could be pre-zygotic, that is, an accumulation of maternal transcripts in the forming oocytes [54] indicating the involvement of wnt signaling in *Botryllus* female germ cell proliferation and migration and not in body patterning.

### Over-activation of Wnt signaling results in ectopic secondary bud formation

Previous studies on flatworms and other regenerative species [7,50,55] has shown that over-activation of the canonical Wnt pathway will turn a non-regenerating tissue into a regenerating epithelia [55-59]. In *Botryllus*, the location of the buds is stereotyped and the presence of ectopic budlets suggests that over-activation on Wnt signaling may induce different regions of the epithelium to become competent for budding. However, this was only seen in the first generation, as budding was abolished in the *N* + 2 generation.

We hypothesize that these observations are due to the downstream effects of Wnt over-activation. In many organisms, axis formation is initiated within organizer regions. If Wnt is responsible for an anterior organizer formation [21,37,60], it may be that an increase in activity could form ectopic anterior organizer. Although the presence of urochordates organizer genes has been debated [61,62], the lack of a clear expression pattern of ascidian organizer-type genes has not been characterized yet. We therefore may only speculate that elevated levels of ectopic anterior inhibition coupled with an elevated level of anterior activation in the budlet apical tip may provide an explanation for the formation of ectopic budlets in drug-treated animals.

### Wnt signaling is necessary for A/P axis determination during blastogenesis

In order to study the effect of wnt during the onset of blastogenesis, colonies were incubated with wnt agonists (APL and LiCl) and antagonists inhibitors (IWP and IWR) [46,63,64], at take-over (stage 11/8/6), when the secondary bud starts to form. While antagonists gave variable and not consistent results (not shown), we observed multiple phenotypes using wnt agonists that reflected a role of the Wnt pathway in the body patterning of *B. schlosseri*.

Several studies have been previously demonstrated how chemical blocking of GSK-3β activity by either paullones or lithium ions leads to a severe alteration of the main body patterning by the over-activation of the Wnt pathway [10,14,55,65,66]. In line with those previous reports, treatments with both APL and LiCl clearly generated phenotypes resulting in body patterning alterations, tissue malformations, and organs misplacement. These phenotypes started to appear only during the second blastogenic cycle (*N* + 1) post incubation, indicating that the tissue affected by the drug was the peribranchial epithelium of the primary bud. The different orientation of the main axis of the primary buds (at stage *N* + 1) can be explained in terms of the anterior and posterior axis organization. Earlier studies showed that the first visual cue of A/P polarization is the skewing of a symmetrical budlet toward the anterior end of the parental bud [29]. Our data suggest that over-activation of Wnt may lead to the progression of the initial asymmetric expansion of the vesicle, blocking or altering the normal rotation and elongation of the budlet along the A/P axes. In fact, in untreated animals, the budlet polarity starts to be shaped in anterior and posterior with a specific patterning that produces an anterior axis inhibition in the posterior side and vice versa. After drug treatments, the posterior axis inhibition level should fall below a threshold value such as a new anterior budlet organizer would arise. This should result in the formation of a polarized new budlet with a strong expression of *wnt2B* localized at the budlet initiation site.

We should mention that not having a *B. schlosseri* anterior or posterior marker and not being able to see the loss of one of those markers upon treatments, it is difficult to confirm defects on A/P axis formation. On the other side, after Wnt agonist incubation, we observed no elongation of the primary bud along the A/P axis, nor rotation of the secondary bud. Being those developmental movements regulated by the Wnt signaling, we are allowed to speculate that changes of the A/P axis of the budlet from being parallel to perpendicular to that of the primary bud are direct evidence of an alteration of the Wnt pathway.

### Wnt signaling perturbation results in axis duplication and cell polarity

In agreement with previous studies on Wnt signaling pathway perturbation [2,55,67], our findings reaffirm the role of Wnt in regulating the body axis specification. Histological sections show a bifurcating budlet during the *N* + 1 blastogenic cycle and zooids with one atrial and two oral siphons at *N* + 2 cycle. It is not however clear why this phenotype was not ubiquitous within the whole colonies but usually affected only 20% of the zooids. Interestingly, a duplicated body axis may be indicative of the formation of second organizer region during early development. Therefore, observing a duplicated axis within drug-treated animals could be an evidence that an organizer-like region does exist during blastogenesis and that its formation is regulated by Wnt signaling. Overall, the entire colony was altered by the treatments clearly showing rounded zooids as well as multi-siphoned individuals confirming a strong impact on the body patterning throughout the three blastogenic cycles studied.

Using a chemical approach may lead the reader to argue that the phenotypes obtained were results of artifacts and therefore not real. Interestingly, we could compare results from treatments with two different drugs known to be Wnt agonist and therefore validate the approach by observing identical phenotypes. Since our treatment was ‘global’, all cells in the treated animals were accessible to paullones or lithium. This could likely result in abnormal numbers of cells being activated and likely entering first a proliferation phase, followed by terminal differentiation, resulting in an abnormal tissue organization. Ultrastructural findings of different cell shapes between the peribranchial epithelium of control and drug-treated budlets may lead to the hypothesis that the PCP pathway is also involved in the asexual development of *B. schlosseri*. However, the cell ultrastructure did not show any significant marks of polarity. We did not notice any reorganization of cell’s tight junctions nor presence of cilia or changes in the distribution of the nucleus and Golgi complex of all features of a proper planar cell polarity. The change of shape from a polarized oval cell to a rounded cell might be interpreted as a shrinkage effect more than a change in polarity. On the other end, the involvement of the GSK3β complex in the non-canonical wnt/PCP pathway is still controversial, [68-70] so we do not completely exclude that in the developing bud of Botryllus, canonical and non-canonical pathway may co-exist.

## Conclusions

Our results provide evidence that altering the Wnt signaling pathway by blocking the GSK-3β complex induces a disruption of the main A/P polarity and a consequent asymmetric development during the asexual cycle of *B. schlosseri*. Unfortunately, we made multiple attempts to verify β-catenin translocation to the nucleus following agonist treatment, but there is no *Botryllus* specific antibody, and our results with other commercially available probes were inconclusive. We therefore cannot exclude the hypothesis that the observed phenotypes were due to activation of the non-canonical Wnt pathway.

Previous reports describe the ligands Wnt11, Wnt4, and Wnt6 being non-canonical [6,8,11,17,71-75]. With few exceptions [76], Wnt5A is also considered a non-canonical Wnt ligand [9,11,77] and its role in lower metazoans has been shown to be crucial for embryonic development [11,16,78,79]. Interestingly, in *B. schlosseri*, *wnt5A* mRNA was exclusively detected in the germline precursors, thus cannot be considered to be involved in body patterning mechanisms. In contrast, among the canonical ligands [2,7,55,77,80], *wnt9A* and *wnt2B* do show expression in the developing budlet of *B. schlosseri* leading us to the hypothesis that the canonical Wnt pathway may have a key role in body patterning and axial polarity during non-embryonic development. This is further confirmed by the high expression pattern of *wnt2B* in budlets of drug-treated colonies.

Many studies have suggested that Wnt pathway may have been instrumental in the evolutionary origin of multicellular animals. Given the fundamental importance of the cellular information mediated by the wnt signaling, it is not surprising that the role of wnt is conserved across phyla during embryogenesis and also during post-embryonic developmental processes, including metamorphosis, regeneration, and asexual reproduction.

## Additional files

Additional file 1: Table S1.
*Botryllus schlosseri wnt* genes sequences and their similar orthologs of other species. Among several *B. schlosseri wnt* genes, four sequences show strong similarities (*e*-value <1*e* ^−40^) with orthologs in other species. The sequences were identified by using a *de novo* assembled transcriptome database (http://octopus.obs-vlfr.fr/public/botryllus/blast_botryllus.php). Additional file 2: Figure S1. Diagram summarizing the phenotypes obtained by drug treatments and their kinetic. In order to study the effect of Wnt during the onset of blastogenesis, colonies were incubated with Wnt agonists and antagonists inhibitors at take-over (stage 11/8/6), when the secondary bud starts to form. While antagonists did not give any reliable results (not shown), we observed multiple phenotypes using LiCl and APL that reflected a role of the Wnt pathway in the body patterning of *Botryllus schlosseri*. Each color represents a generation sketched on the three bottom cartoons. Solid lines symbolize a normal development, and broken lines stand for an abnormal development. During the *N* cycle, at the beginning of the drug incubations, zooids (light blue) and primary buds (turquoise) are not affected by the treatments (solid lines). Only the secondary bud (dark blue) starts to show signs of symmetry breaking toward the end of the cycle (broken line). The same generation (dark blue) looks rounded at *N* + 1 cycle when the former primary bud, now adult zooid (turquoise), has an abnormal morphology. Here, the new generation (dark red) starts developing ectopically already with a rounded shape that will be inherited at *N* + 2 cycle. At take-over (X), budlets (dark blue) become adult zooids showing A/P malformations ranging from double axes to rounded shape with misplaced organs.

## Abbreviations

BLAST: Basic Local Alignment Search Tool
DMSO: dimethyl sulfoxide
FSW: filtered sea water
GSK-3β: glycogen synthase kinase 3 beta
JTT: Jones-Taylor-Thorton mathematical method.
PCR: polymerase chain reaction
