# Supplementary material for: Wnt affects symmetry and morphogenesis during post-embryonic development in colonial chordates
Source: EvoDevo. 2015 May 1;6:17. doi: 10.1186/s13227-015-0009-3 (PMC4499891; doi:10.1186/s13227-015-0009-3)
Supplement: Additional file 1: Table S1. — Botryllus schlosseri wnt genes sequences and their similar orthologs of other species. Among several B. schlosseri wnt genes, four sequences show strong similarities (e-value <1e −40) with orthologs in other species. The sequences were identified by using a de novo assembled transcriptome database (http://octopus.obs-vlfr.fr/public/botryllus/blast_botryllus.php). [file 13227_2015_9_MOESM1_ESM.doc]

**Additional file 2: Table s1.** *Botryllus schlosseri wnt* genes sequences and their similar orthologs of other species.

| **NAME** | **mRNA accession number (Bs_trinity_v1.0)** | **1st hit/Sequence ID** | **E-value** |
| --- | --- | --- | --- |
| *wnt2B* | comp556459_c5_seq5 len=2180 | PREDICTED: protein Wnt-2b [Sarcophilus harrisii]  Sequence ID: ref|XP_003769833.1| | 5E-89 |
| *wnt7B* | comp561241_c1_seq5 len=1039 | HrWnt-7 [Halocynthia roretzi]  Sequence ID: dbj|BAB63964.1| | 6E-49 |
| *wnt9B* | comp566883_c1_seq22 len=3087 | PREDICTED: uncharacterized protein LOC100175210 [Ciona intestinalis]  Sequence ID: ref|XP_009859770.1| | 1E-31 |
| *wnt5A* | comp564602_c4_seq4 len=1015 | ascidian homolog of wnt-5 [Halocynthia roretzi]  Sequence ID: dbj|BAB88819.1| | 1E-99 |
